# Pharmacomicrobiomics in inflammatory skin diseases: past, present, and the future

**DOI:** 10.3389/fmicb.2025.1745985

**Published:** 2026-01-14

**Authors:** Juna Khang, Rebeca Martinez, Katherine Brag, Jean S. McGee

**Affiliations:** 1 Department of Dermatology, Brigham and Women's Hospital, Boston, MA, United States; 2 Harvard Medical School, Boston, MA, United States; 3 Department of Dermatology, Beth Israel Deaconess Medical Center, Boston, MA, United States

**Keywords:** biomarker, gut micriobiome, inflammatory disease, pharmacomicrobiomics, therapeutic

## Abstract

This mini review article focuses on pharmacomicrobiomics, or the study of how the composition and activity of microorganisms in the body, in particular in the gut, impact drug pharmacokinetics and pharmacodynamics. This evolving field has profound implications for personalized medicine in the management of chronic inflammatory diseases. Particularly in dermatology, patient response to an expanding collection of biologic and small molecule inhibitor therapies coming out on the market remains unpredictable. The decision to start which therapy depends on physician preference, rather than based on what is expected to be the treatment response of each individual. This therapeutic uncertainty leads to sequential treatment failures, increased patient morbidity, and substantial healthcare expenditure. This mini-review synthesizes the evidence surrounding the gut microbiome as a predictive biomarker for therapeutic response in inflammatory skin diseases. We will examine the past use of pharmacomicrobiomics in oncology, where gut microbial signatures were found to predict response to immunotherapy in melanoma. We then analyze the present, focusing on the robust translational models from inflammatory bowel disease (IBD) and rheumatoid arthritis (RA), and the established gut dysbiosis in dermatologic conditions such as psoriasis and hidradenitis suppurativa (HS). Finally, we consider the future, discussing the potential for microbiome-based diagnostics to guide therapy selection for chronic inflammatory skin diseases.

## 1 Introduction

The human gut microbiome acts as a complex metabolic organ that significantly influences host physiology, particularly immune system function (Lee and Mazmanian, 2010; O’Hara and Shanahan, 2006). Comprising trillions of bacteria, the gut microbiome provides extensive enzymatic and metabolic capabilities that the human host cannot perform independently (Gilbert et al., 2018; Bäckhed, 2012). An emergent field, pharmacomicrobiomics studies how inter-individual variations in this microbial ecosystem affect drug metabolism and efficacy, as well as disease pathogenesis and immune dysregulation (Wilson and Nicholson, 2017; Zhang et al., 2018). This is highly relevant to chronic inflammatory skin diseases such as psoriasis and hidradenitis suppurativa, which are characterized by complex immune dysregulation. While the management of these inflammatory skin diseases have been revolutionized by targeted biologic and small-molecule inhibitors that interfere with specific cytokines and signaling pathways, such as Tumor Necrosis Factor-alpha (TNF-*α*), specific interleukins (IL-17, IL-23, IL-4, IL-13), and the Janus kinase (JAK) pathway, a substantial portion of patients with psoriasis exhibit a primary lack of response (Piragine et al., 2022; Pina Vegas et al., 2022; Shin et al., 2023). The effectiveness of current biologics for HS is even lower compared to other inflammatory diseases, with moderate effectiveness of about 50% of patients achieving a clinical response at 12–16 weeks for phase III trials of the three FDA-approved biologics (adalimumab, secukinumab, and bimekizumab) for moderate-to-severe HS (Kimball et al., 2016, 2023, 2024; Tešanović Perković et al., 2025; Krueger et al., 2024). Currently, the selection of biologic treatment for HS is largely empirical, guided by physician preference and comorbidity profiles rather than by validated predictive biomarkers (Ring et al., 2022). This trial-and-error approach contributes to prolonged disease activity, significant patient burden, and high costs for healthcare systems (Krueger et al., 2024).

In contrast to a trial-and-error empiric approach to treatment of inflammatory skin diseases, microbiome-directed therapies have the potential to restore microbial balance and modulate dysregulated inflammatory pathways. Dysbiosis of the gut-skin axis is implicated in the pathogenesis and progression of these diseases, with altered microbial composition contributing to immune dysregulation and impaired skin barrier function (Memariani and Memariani, 2025; Kapoor et al., 2022; Chen et al., 2020; Buhaș et al., 2022; Aguwa et al., 2023). In this review, we will trace the field of pharmacomicrobiomics from its early associations to current mechanistic and translational insights across multiple medical specialties, and finally towards future treatment response improvement in inflammatory dermatologic conditions.

## 2 The past: principles from oncology

The principle that the gut microbiome can serve as a predictive biomarker for systemic therapy was first robustly demonstrated in oncology, particularly in the context of immune checkpoint inhibitor (ICI) therapy for metastatic melanoma. Multiple landmark studies have shown that the structure and function of the gut microbiome are significant determinants of clinical response to anti–programmed cell death protein 1 (PD-1) immunotherapy. Responders to ICIs consistently exhibit greater alpha diversity and a relative enrichment of species within the Ruminococcaceae family, including *Faecalibacterium prausnitzii*, as well as *Bifidobacterium longum* and *Collinsella aerofaciens* (Limeta et al., 2020; Barragan-Carrillo et al., 2025; Lim et al., 2024; Lu et al., 2022; Matson et al., 2018; Gopalakrishnan et al., 2018). In contrast, non-responders tend to have a higher relative abundance of the Bacteroidales order and reduced microbial diversity. Specifically, metagenomic and metatranscriptomic studies have demonstrated that responders show upregulated anabolic pathways, particularly those involved in short-chain fatty acid (SCFA) biosynthesis, which enhance gut barrier integrity and promote anti-inflammatory immune response that improve systemic and intratumoral immunity through the differentiation and activation of effector T cells and well as the expansion of memory T cells (Peters et al., 2019; Bouferraa et al., 2023; Witt et al., 2023; Simpson et al., 2022). Non-responders are characterized by a higher relative abundance of Bacteroidales and enrichment in catabolic pathways, including those for carbohydrate degradation and fermentation, which may contribute to a less favorable immunologic environment for checkpoint inhibitor efficacy (Gopalakrishnan et al., 2018; Wind et al., 2020). In summary, the composition and functional capacity of the gut microbiome are significant determinants of clinical response to immune checkpoint inhibitors in metastatic melanoma, with specifically high diversity and SCFA production serving as predictive biomarkers that can guide systemic therapy selection (see Table 1).

**Table 1:** Gut microbiome findings in inflammatory dermatoses.

Disease	Key microbiome features	Immune dysregulation	Potential predictive biomarkers	Evidence strength	Critical research gaps
Psoriasis	↓ Alpha diversity ↓ SCFA producers (*Faecalibacterium*, *Ruminococcus*) ↑ Pathobionts (*Prevotella*, *Streptococcus*)	Th17/IL-23 axis activation Impaired Treg function Gut barrier disruption	↑ SCFA producers predict IL-17/23 inhibitor response Taurine/hypotaurine pathway enrichment associated with remission	*Moderate* - Multiple cohort studies - Associations with treatment response - Limited prospective validation	- Large prospective trials validating baseline microbiome for biologic selection - Mechanistic studies linking specific taxa to Th17 activation - Multi-omic integration (metabolomics, immunophenotyping)
Atopic dermatitis	Early-life dysbiosis: ↓ *Bifidobacterium* ↓ *Lactobacillus* ↑ *Clostridium* ↑ Enterobacteriaceae	Th2 polarization ↑ IL-4, IL-13 IgE-mediated inflammation Barrier dysfunction	Low *Bifidobacterium*/*Lactobacillus* in infancy predicts AD development Gut barrier biomarkers (I-FABP, Reg3A) correlate with severity	*Moderate-high* - Large prospective birth cohorts - Probiotic trials show prevention efficacy - Predictive models for AD risk	- Biomarkers predicting biologic response in established AD - Optimal timing/strains for probiotic prophylaxis - Integration with genetic risk scores
Hidradenitis suppurativa	Gut: ↑ *Ruminococcus gnavus* ↓ Commensal diversity Skin: ↑ Anaerobes (*Prevotella*, *Porphyromonas*, *Fusobacterium*)	Innate immune activation TNF-α, IL-17, IL-1β driven inflammation Strong IBD association	*Undefined* No validated predictive biomarkers for biologic response	*Low* - Descriptive microbiome studies - No pharmacomicrobiomics trials - Epidemiologic IBD association	- Baseline microbiome profiling predicting anti-TNF/IL-17/IL-1 response - Skin vs. gut microbiome contributions - Role of anaerobic skin bacteria in systemic inflammation - Mechanistic FMT or targeted antibiotic studies

## 3 The present: building the case in dermatology through parallel inflammatory models

Current research is actively translating the principles established in oncology, in which the gut microbiome serves as a predictive biomarker for systemic therapy, particularly ICI therapy, to the broader spectrum of chronic inflammatory diseases. IBD and RA provide valuable translational models for dermatology due to their shared immune-mediated inflammatory mechanisms, similar therapeutic classes (anti-TNF, IL-17/23 inhibitors, and established gut-systemic immune axis crosstalk).

Advanced translational models in IBD have demonstrated that baseline gut microbiome structure and function can predict response to biologic therapies. Multiple cohort studies consistently identify three key predictive features: (1) increased baseline alpha microbial diversity, (2) increased abundance of SCFA-producing bacteria (e.g., *Faecalibacterium*, *Roseburia*, and *Ruminococcus*), and (3) depletion of pathobionts (e.g., *Escherichia*, *Shigella*, and *Collinsella*) associated with favorable clinical and endoscopic remission rates across anti-TNF agents, vedolizumab, and ustekinumab (Pu et al., 2022; Aden et al., 2019; Zheng et al., 2025; Palmieri et al., 2024; Lee et al., 2021; Caenepeel et al., 2024).

Specifically, for anti-TNF agents for IBD, patients with a dysbiotic Bacteroides 2 (Bact2) enterotype have higher remission rates compared to those treated with vedolizumab, and anti-TNF therapy is associated with a shift toward a more eubiotic microbiome, increased microbial load (i.e., total number of bacterial cells per gram of stool), and butyrogen abundance (Pu et al., 2022; Lee et al., 2021). Vedolizumab responders show enrichment of SCFA-producing genera and specific taxa from the Barnesiellaceae and Lachnospiraceae families (Zheng et al., 2025). Ustekinumab responders are characterized by higher baseline *Faecalibacterium* and lower *Escherichia* and *Shigella* (Lee et al., 2021; Radhakrishnan et al., 2022). Predictive models integrating microbiome features (i.e., diversity, key taxa, and metabolic function), stool characteristics (i.e., calprotectin and moisture), and clinical data achieve robust accuracy (AUC up to 0.89) for forecasting biologic response (Pu et al., 2022; Aden et al., 2019; Palmieri et al., 2024). These findings support the clinical utility of microbiome profiling for personalized biologic selection and highlight the potential for microbiome modulation strategies to enhance therapeutic efficacy in IBD.

Similary, RA serves as another analog for how specific gut microbiome alterations drive systemic immune dysregulation. Evidence supports the role of gut dysbiosis in RA development and progression, characterized by reduced microbial diversity and expansion of pro-inflammatory taxa, notably *Prevotella copri*, which is enriched in new-onset RA and in individuals at high risk for RA (Korzeniowska and Bryl, 2024; Alpizar-Rodriguez et al., 2019; Seifert et al., 2023). RA’s mechanistic relevance to dermatology lies in three key pathways: (1) *P. copri* disrupts intestinal barrier integrity, leading to translocation of microbial products (e.g., lipopolysaccharide) that promote systemic inflammation and immune activation (Longo et al., 2024; Brandl et al., 2021; Qi et al., 2024; Zaiss et al., 2021; Romero-Figueroa et al., 2023); (2) It stimulates dendritic cells to produce IL-6 and IL-23, which promote Th17 cell differentiation, a pathway shared with psoriasis and HS (Pianta et al., 2017; Maeda et al., 2016; Chen et al., 2025); (3) It presents antigenic peptides that activate autoreactive T cells through molecular mimicry (Korzeniowska and Bryl, 2024; Seifert et al., 2023; Longo et al., 2024).

Direct causal evidence comes from fecal microbiota transplantation studies: transplantation studies in which transplantation of microbiota from anti-CCP positive, preclinical RA subjects into mice resulted in reduced microbial diversity, increased intestinal permeability, Th17 expansion, and heightened arthritis severity (Chen et al., 2025; Luo et al., 2023; Belvončíková et al., 2025). These findings demonstrate that gut dysbiosis, particularly expansion of *P. copri*, can trigger mucosal immune activation and barrier dysfunction, driving progression from preclinical autoimmunity to overt disease, a mechanism highly relevant to the gut-skin axis in dermatologic conditions.

Robust pharmacomicrobiomics research in IBD and RA provides a direct and compelling parallel for dermatology, as they share therapeutic targets (TNF-α, IL-17, and IL-23) and similar patterns of gut dysbiosis. For example, similar to that of RA, gut dysbiosis in psoriasis is characterized by reduced microbial diversity, depletion of SCFA producers (*Facalibacterium*, *Ruminococcus*, and *Lachnospira*), and increased abundance of pathobionts (*Prevotella* and *Steptococcus*) (Xue et al., 2025; Polak et al., 2021; Fallahi et al., 2025). Multiple studies using 16S rRNA gene and shotgun metagenomic sequencing have consistently demonstrated lower alpha diversity and richness in the gut microbiota of psoriasis patients compared to healthy controls, with this reduction correlating with disease severity (Todberg et al., 2022; Schade et al., 2022; Olejniczak-Staruch et al., 2021; Hidalgo-Cantabrana et al., 2019). In the mechanistic link to the Th17/IL-23 axis, depletion of SCFA producers reduces butyrate levels, impairing regulatory T cell function and gut barrier integrity. This promotes systemic inflammation and Th17 activation, directly contributing to psoriatic plaque formation and reduced response to therapy (Memariani and Memariani, 2025; Buhaș et al., 2022; Kreouzi et al., 2025; De Pessemier et al., 2021).

Just as in gastroenterology and rheumatology, the clinical challenge of individualized biologic selection for each patient in dermatology persists. Biologic therapies targeting TNF, IL-17, and IL-23 pathways modulate gut microbiota composition and function in patients with psoriasis and psoriatic arthritis by shifting microbial diversity, altering the abundance of key taxa, and impacting microbial metabolic pathways. IL-17 and IL-23 inhibitors are associated with dynamic changes in gut microbiota, including increased alpha diversity and restoration of beneficial taxa such as Ruminococcaceae and *Phocaeicola*, as well as functional upregulation of carbohydrate-active enzymes and metabolic pathways related to amino acid and taurine/hypotaurine biosynthesis (Liu et al., 2025). TNF inhibitors also induce shifts in gut microbiota, but the patterns differ from those seen with IL-17/23 blockade (Manasson et al., 2020).

Microbiome alterations may serve as predictive biomarkers for biologic response in psoriasis. Baseline gut microbiome composition and functional gene profiles have been associated with treatment outcomes, with specific taxa and metabolic pathways enriched in responders versus non-responders to IL-17 and IL-23 inhibitors. For example, increased abundance of SCFA producers and certain metabolic pathways (e.g., taurine/hypotaurine) are linked to favorable response (Yeh et al., 2019; Huang et al., 2023; Du et al., 2023). Conversely, persistent dysbiosis or overgrowth of pathobionts can predispose to infections or paradoxical inflammation (Vassilopoulos et al., 2022; Fang et al., 2024).

Alterations in the gut microbiome are also being increasingly recognized as a key factor in the pathogenesis of atopic dermatitis (AD), particularly through their impact on immune system development and deviation toward T-helper 2 (Th2) responses. Early-life gut microbiota composition and diversity are critical determinants of allergic disease development, with specific taxa and reduced diversity serving as predictive markers for AD and related conditions. Specifically, multiple large prospective cohort studies demonstrate that infants who later develop AD often exhibit delayed or aberrant gut microbiota maturation, with lower levels of *Bifidobacterium* and *Lactobacillus* and higher levels of *Clostridium* and Enterobacteriaceae during the first year of life (Ta et al., 2020; Imoto et al., 2025; Hoskinson et al., 2023; Donald and Finlay, 2023). This dysbiotic environment favors the activation of dendritic cells and the release of cytokines that promote Th2 polarization, while suppressing T-helper 1 (Th1) responses. The resulting immune deviation is marked by increased production of Th2 cytokines (e.g., IL-4, IL-13), which drive IgE-mediated allergic inflammation and the development of atopic diseases such as AD (Imoto et al., 2025; Bellinghausen et al., 2022; Pascal et al., 2018). Biomarkers of gut barrier dysfunction and altered gut-derived metabolites, including intestinal fatty acid binding protein (I-FABP), Regenerating islet-derived protein 3 alpha (Reg3A), SCFAs such as caproic and isocaproic acid, and indoxyl, have also been correlated with AD severity (Song et al., 2016; Reddel et al., 2019; Ma et al., 2024; Blicharz et al., 2025). Collectively, the evidence supports a model in which early-life gut microbiota composition, particularly the balance of *Bifidobacterium*, *Lactobacillus*, *Clostridium*, and Enterobacteriaceae, plays a pivotal role in immune development and the subsequent risk of AD. While biomarkers are largely risk-predictive rather than drug-response predictive, they highlight the potential for therapeutic improvement in risk and severity. For example, early probiotic intervention with *Bifidobacterium* and *Lactobacillus* have been shown to decrease AD risk and severity, particularly in predisposed children (Tang et al., 2025; Chang et al., 2016). This represents evidence of microbiome modulation improving dermatologic outcomes, although predictive biomarkers for targeted biologic therapy in AD remains underdeveloped.

Pharmacomicrobiomics is particularly relevant in hidradenitis suppurativa, a systemic inflammatory condition with profound inflammatory burden and a strong association with IBD. The association between HS and IBD is robust and bidirectional, suggesting a common gut-skin pathogenesis. Multiple large-scale epidemiological studies have demonstrated that patients with HS have significantly increased odds of developing both Crohn disease and ulcerative colitis compared to the general population (Nguyen et al., 2021; Lönndahl et al., 2025; Egeberg et al., 2017; Chen and Chi, 2019). Current evidence demonstrates that HS is characterized by significant dysbiosis of both the skin and gut microbiome, with a predominance of anaerobic bacteria such as *Prevotella*, *Porphyromonas*, *Fusobacterium*, and *Parvimonas* in lesional skin and tunnels, and altered gut microbial profiles with increased *Ruminococcus gnavus* and decreased commensal diversity (Williams et al., 2024; Williams et al., 2021; Soto-Moreno et al., 2025; McCarthy et al., 2022; Guet-Revillet et al., 2017). While antimicrobial therapy is a mainstay of HS management and clinical improvement is often observed with antibiotics, the specific interactions between the microbiome and pharmacologic agents, such as how microbial composition modulates drug efficacy, metabolism, or resistance, have not been systematically characterized in HS. Furthermore, FDA-approved biologics are only moderately effective, and the nature of treatment selection is largely empirical. Thus, pharmacomicrobiomics is a logical and promising area of investigation to understand the highly variable patient responses in HS. Determining if a patient’s gut microbiome profile is similar to that in IBD (high anaerobes, low diversity) could stratify patients who may benefit more from TNF-inhibition versus IL-1 or IL-17 blockade. There remains a need to clinically validate these associations as predictive biomarkers with future research of large-scale prospective studies with mechanistic validation.

The gut-skin axis should also be considered in dermatological applications of pharmacomicrobiomics. The skin microbiome directly influences local immune responses and barrier function in psoriasis, AD, and HS. Gut-derived SCFAs and tryptophan metabolites modulate systemic immune responses that affect skin inflammation, while skin barrier disruption and local inflammation may influence gut permeability and microbiome composition through systemic immune activation (Memariani and Memariani, 2025; Aguwa et al., 2023; De Pessemier et al., 2021). Both gut and skin microbiomes may independently or synergistically identify treatment response to systemic biologic therapies. For example, enrichment of SCFA-producing gut bacteria along with restoration of commensal skin microbes could identity optimal responders to IL-17 inhibition. Future pharmacomicrobiomics research should therefore consider integration of microbiome profiling from different sources.

## 4 The future: towards targeted therapeutic modulation

The future trajectory of pharmacomicrobiomics in dermatology lies in the advancement of microbiome-based diagnostics and therapeutics to predict and optimize a patient’s response to specific therapy. The development of validated microbiome-based diagnostics and its integration into clinical practice may enable a more personalized approach to biologic selection and adjunctive therapy in inflammatory skin conditions such as HS, psoriasis, and AD. Analysis of a patient’s stool sample via high-throughput sequencing methods like 16S rRNA gene sequencing (for taxonomic profiling) or shotgun metagenomic sequencing (for taxonomic and functional profiling) could identify microbial signatures predictive of response to specific classes of drugs (e.g., TNF-inhibitors, IL-17 inhibitors, and JAK inhibitors). For example, a future diagnostic test for a psoriasis patient could analyze a stool sample to quantify the baseline abundance of SCFA producers, as a gut microbiome rich in taxa such as *Faecalibacterium* might predict a favorable response to an IL-17 inhibitor. For AD, diagnostics might focus on infants, analyzing early-life gut microbiota for deficiencies in *Bifidobacterium* and *Lactobacillus*, thereby identifying high-risk individuals who may benefit from early prophylactic interventions. In HS, characterizing individual dysbiotic patterns and immune activation profiles would allow dermatologists to choose specific biologic agents targeting relevant individualized inflammatory pathways. For instance, patients with pronounced microbial-driven innate immune activation may respond differently to TNF-α inhibitors or IL-17/IL-1 blockade, though direct evidence linking microbiome diagnostics to biologic selection in HS is currently lacking. Such a diagnostic tool would allow dermatologists to stratify patients and select a first-line therapy with a much higher probability of success. However, the path to implementation is not straightforward. Significant barriers include the high cost of sequencing, the lack of standardized sampling protocols, unclear regulatory frameworks for microbiome diagnostics, significant intra-individual temporal variation in gut microbiome, and finally, clinical validation in prospective, randomized clinical trials.

Therapeutics tailored to individual microbiome profiles show an area of promise in dermatology. Strategies to edit a non-responder microbiome signature into a responder signature encompass several approaches. Fecal Microbiota Transplantation (FMT) presents a powerful option to completely reset a dysbiotic gut microbiome, in cases such as severe, refractory HS. However, FMT does have regulatory hurdles, safety concerns of pathogen transmission, and limited mechanistic understanding of optimal donor selection and engraftment. Clinical trials are needed to define which patient populations and phenotypes would most benefit.

Probiotics and prebiotics have also demonstrated the ability to increase SCFA production and favorably modulate immune pathways relevant to IL-17 signaling. Specific probiotic strains that enhance SCFA levels have been shown in preclinical models to suppress IL-17 and IL-23, ameliorating psoriatic inflammation (Buhaș et al., 2023). Early probiotic and prebiotic interventions can also reduce the incidence and severity of AD, especially in genetically predisposed children. Probiotic supplementation with *Bifidobacterium* and *Lactobacillus* strains, as well as prebiotics that promote their growth, have been shown to decrease the risk of AD by up to 30% and improve clinical severity scores (Tang et al., 2025; Chang et al., 2016). Factors such as diet, antibiotic use, and birth mode significantly influence infant gut microbiota composition and should be considered when designing targeted prophylactic strategies. Postbiotics represent an even more targeted approach, involving the administration of microbial metabolites (such as SCFAs) that directly exert anti-inflammatory effects and support epithelial barrier function, bypassing the need for live bacteria (Memariani and Memariani, 2025). This strategy is attractive for its safety profile but requires deeper mechanistic understanding of gut microbiome dysbiosis and pharmacomicrobiomics therapy in HS, as clinical translation requires strain-specific validation and optimization of dosing and delivery.

Despite promising advances, there is a lack of large-scale, clinic trials evaluating microbiome-targeted adjunctive therapies in chronic inflammatory skin conditions, particularly HS. Many current studies rely on small sample sizes and cross-sectional designs, limiting causal inference. Variation in sequencing methodologies (for example, 16S vs. shotgun metagenomic) and sample processing creates challenges for cross-study validation. Most dermatologic studies rely on taxonomic data alone; therefore, functional metagenomics measuring metabolic pathways and gene expression may provide more robust biomarkers. Mechanistic studies are also required to elucidate the causal relationships between microbial dysbiosis, immune activation, and disease progression, and to guide the development of precision interventions. The field is moving toward a more personalized, microbiome-informed approach, but further research is essential to translate these insights into improved clinical outcomes.

## 5 Conclusion

Pharmacomicrobiomics has shown promise in the management of chronic, inflammatory skin diseases. The current empirical approach to selecting systemic therapies for severe inflammatory skin diseases is suboptimal for both patients and healthcare systems. The foundational work in melanoma has proven that the gut microbiome can serve as a powerful predictive biomarker for treatment response. Guided by robust translational models in IBD and RA, current research is now actively investigating this potential in psoriasis, HS, and AD. The future integration of microbiome diagnostics and therapeutics has the potential to deliver personalized and effective care, minimizing patient suffering from treatment failures and optimizing the use of high-cost therapies, by treating a patient’s unique microbial landscape. The field of pharmacomicrobiomics shows varying levels of promise for translation in dermatology: (1) AD shows the strongest evidence for microbiome modulation (early probiotic intervention) improving outcomes, with validated early-life risk biomarkers; (2) psoriasis demonstrates emerging associations between baseline microbiome composition and biologic response, requiring prospective validation; and (3) HS represents the greatest unmet need, with established dysbiosis but no validated pharmacomicrobiomics biomarkers despite strong IBD overlap and poor treatment response rates.

The integration of microbiome diagnostics and therapeutics has the potential to deliver personalized and effective care, minimizing patient suffering from treatment failures and optimizing the use of high-cost biologic therapies by treating a patient’s unique microbial signature. However, realizing this potential requires: (1) large, adequately powered prospective trials with standardized methodologies; (2) regulatory frameworks and reimbursement pathways for microbiome diagnostics; (3) mechanistic validation of causal relationships between dysbiosis and treatment resistance; (4) cost-effectiveness demonstration in real-world settings; and (5) development of clinically feasible, simplified assays suitable for routine practice.
